# National recommendations for the management of children and young people with IgA vasculitis: a best available evidence, group agreement-based approach

**DOI:** 10.1136/archdischild-2024-327364

**Published:** 2024-10-08

**Authors:** Louise Oni, Caroline Platt, Matko Marlais, Liza McCann, Farah Barakat, Markus Hesseling, Hannah Cottis, Sue Protheroe, Gabrielle Haigh, Kerstin Nott, Julien Marro, Elizabeth King, Jane Kelly, Jill Sussens, Shirley Mulvaney, Thomas Whitby, Iona Morgan, Amita Sharma, Reem Al-Jayyousi, Chee Kay Cheung, Christopher Ng, Anthony David Lander, William Simmons, Charlotte Melling, Rebecca Grandison, Leanne Treitl, Alan D Salama, Jan Dudley

**Affiliations:** 1 Department of Women’s and Children’s Health, University of Liverpool, Liverpool, UK; 2 Department of Paediatric Nephrology, Alder Hey Children’s Hospital, Liverpool; 3 Bristol Renal Unit, Bristol Royal Hospital for Children, Bristol, UK; 4 Department of Paediatric Nephrology, Great Ormond Street Hospital, London, UK; 5 Department of Paediatric Rheumatology, Alder Hey Children’s NHS Foundation Trust, Liverpool, UK; 6 Kings College Hospital NHS Foundation Trust, London, UK; 7 Department of Paediatrics, Children’s health Ireland, Dublin, Ireland; 8 Department of Paediatrics, Royal Devon University Hospital, Devon, UK; 9 Department of Paediatric Gastroenterology, Birmingham Children’s Hospital, Birmingham, UK; 10 Department of Paediatrics, Betsi Cadwaladr Health Board, Wales, UK; 11 Department of Paediatric Rheumatology, Southampton Children’s Hospital, Southampton, UK; 12 University of Liverpool Medical School, Liverpool, UK; 13 Woolton Medical Centre, Liverpool, UK; 14 General Practice, Minchinhampton Surgery, Gloucestershire, UK; 15 Nottingham Children's Hospital, Nottingham University Hospitals NHS Trust, Nottingham, UK; 16 Department of Paediatric Emergency Medicine, Alder Hey Children’s Hospital, Liverpool, UK; 17 General Paediatrics, Alder Hey Children's Hospital, Liverpool, Merseyside, UK; 18 Department of Paediatrics, Royal Hospital for Children, Glasgow, UK; 19 College of Medicine, Baghdad, Iraq; 20 University Hospitals of Leicester NHS Trust, Leicester, UK; 21 Sheffield Children’s Hospital, Sheffield, UK; 22 Surgery, Birmingham Children's Hospital, Birmingham, UK; 23 Department of Paediatric Pathology, Alder Hey Children’s Hospital, Liverpool, UK; 24 Department of Paediatric Surgery, Alder Hey Children’s Hospital, Liverpool, UK; 25 Patient Representative, -, UK; 26 Department of Renal Medicine, UCL Centre for Kidney and Bladder Health, London, UK

**Keywords:** Child Health, Nephrology, Rheumatology

## Abstract

**Objective:**

IgA vasculitis (IgAV) is the most frequently experienced subtype of vasculitis seen in children. Most children fully recover, however, complications including chronic kidney disease are recognised. The aim of this project was to use a best available evidence, group agreement, based approach to develop national recommendations for the initial management of IgAV and its associated complications.

**Methods:**

A fully representative multiprofessional guideline development group (GDG), consisting of 28 members, was formed and met monthly. Graded recommendations were generated using nationally accredited methods, which included a predefined scope, open consultation, systematic literature review, evidence appraisal, review of national or international guidelines and a period of open consultation. Audit measures and research priorities were incorporated.

**Results:**

The IgAV GDG met over a 14-month period. A total of 82 papers were relevant for evidence synthesis. For the initial management, four topic areas were identified with five key questions generating six graded recommendations related to classification, specialist referral and musculoskeletal involvement. For the associated complications, five topic areas with 12 key questions generated 15 graded recommendations covering nephritis, gastrointestinal and testicular involvement, atypical disease and follow-up. Open consultation feedback was incorporated. The guidelines were endorsed by the UK Kidney Association and Royal College of Paediatrics and Child Health and are available online.

**Conclusion:**

Despite IgAV being a rare disease with limited evidence, a national standardised approach to the clinical management for children and young people has been achieved. This should unite approaches to care and act as a foundation for improvement.

**WHAT IS ALREADY KNOWN ON THIS TOPIC**

**WHAT THIS STUDY ADDS**

- A multiprofessional derived national guideline to align the care of IgA vasculitis for children and young people based on a best available evidence and group agreement structured approach.

**HOW THIS STUDY MIGHT AFFECT RESEARCH, PRACTICE OR POLICY**

- A unified approach to enable equity of care for children and young people with IgA vasculitis.
- Nationally accredited guidance that can act as a framework for audit and evidence generation to improve future outcomes of this condition.

## Introduction

 IgA vasculitis is a rare disease, however it is the most frequently experienced subtype of vasculitis in children with an estimated annual incidence of 6.8 to 30 per 100 000 childhood population.^1 2^ Adult-onset disease is ultrarare with an estimated incidence of 0.8–1.8 per 100 000 adult population.^3^ It is classified as a small-vessel vasculitis characterised by IgA1-dominant immune deposits in the vasculature and affected tissues. The condition typically presents as a purpuric, symmetrical, non-blanching rash, predominating on the extensor surfaces of the lower limbs.^4 5^ It is associated mainly with skin involvement together with any of gastrointestinal, musculoskeletal and/or renal involvement (termed IgAV nephritis, IgAVN).^6^ Overall, the disease is self-limiting in most children, however short-term morbidity related to abdominal involvement is seen and recurrent episodes are reported.^7^ In the longer term chronic kidney disease (CKD) is a major consequence of this condition. The incidence of kidney failure in all children presenting with IgAV is consistently reported at 1%–2%,^8 9^ yet it is much higher at around 10% in adult-onset disease. In studies that capture children with biopsy-proven kidney disease, termed IgAV nephritis, the rates of CKD are much higher, especially in patients with poor prognostic features such as impaired baseline kidney function.^10^ Current treatment options for established nephritis rely on broad-spectrum immunosuppressive agents and randomised controlled trial data have shown that the prophylactic use of high-dose corticosteroids at disease onset in all patients does not reduce the risk of developing subsequent nephritis.^11 12^ A lack of a standardised approach to this disease promotes variability in patient care and is a barrier to improving outcomes. Furthermore, the rarity of the disease in the adult population means that extrapolation of evidence has not been feasible. In 2019, an international European-wide collaborative effort—called the Single Hub and Access point for paediatric Rheumatology in Europe (SHARE) initiative—provided a milestone in advancing this condition internationally.^13^

The aim of this project was to use a best available evidence, group agreement, based approach to create national recommendations for the initial management and associated complications of IgAV for children and young people in the United Kingdom.

## Methods

**Figure 1:**
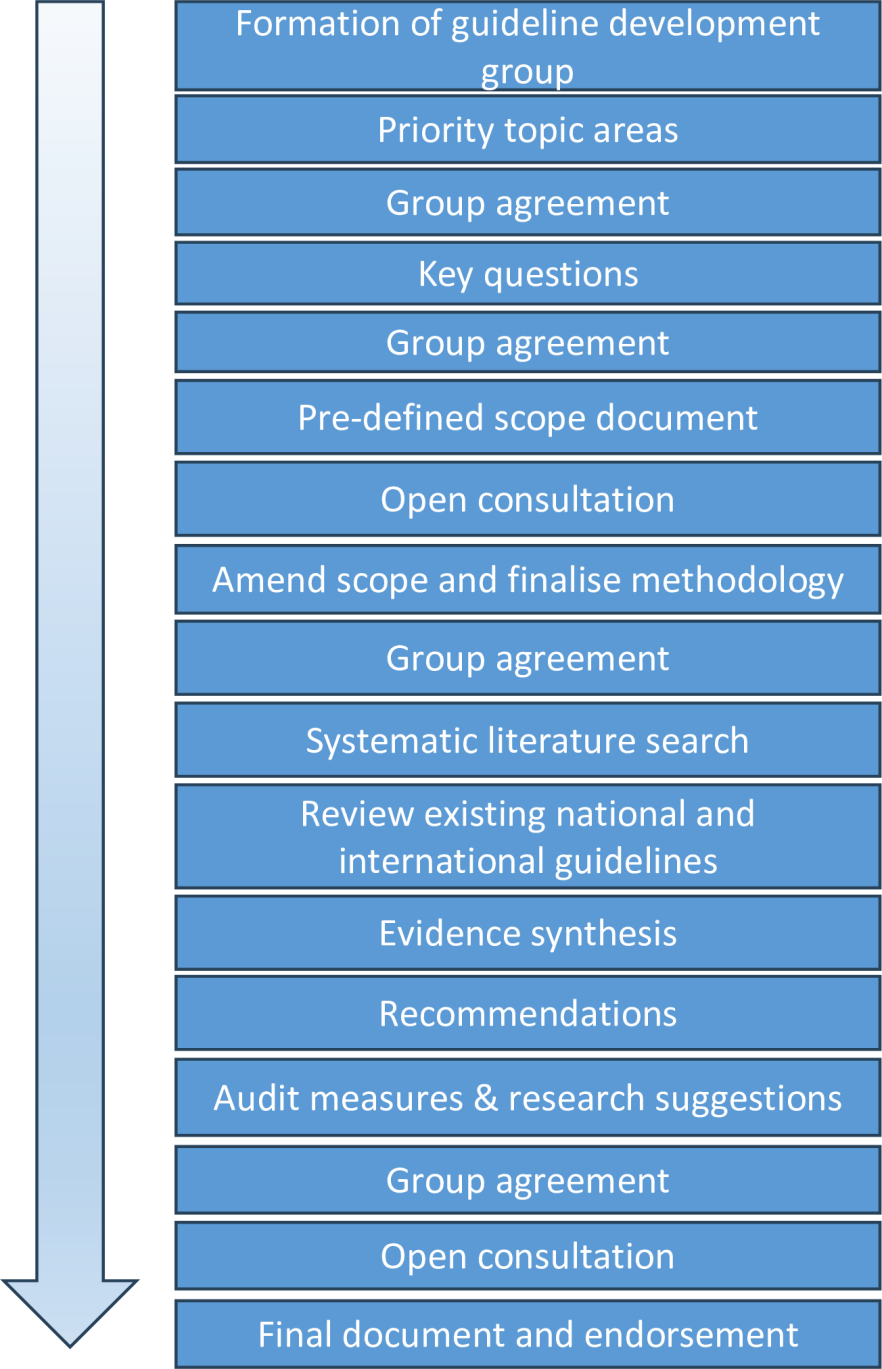
A summary of the methodology used to generate recommendations for a rare disease using a best available evidence and group agreement-based approach.

The methodological approach used a multiprofessional working group, topic areas with priority questions, a predefined scope and evidence synthesis to develop graded recommendations for IgAV (outlined in figure 1).

### Establishing a multiprofessional working group

A multiprofessional guideline development group (termed the IgAV GDG) was established via the Royal College of Paediatrics and Child Health (RCPCH) Research and Evidence team. Members were required to submit an expression of interest and declare any conflicts of interest. The methodology adhered to clinical guideline standards outlined by the RCPCH and the UK Kidney Association (National Institute of Clinical Excellence accredited) as a collaborative venture.

### Developing the scope and key questions

The IgAV GDG defined the topic areas and key questions for the scope document (table 1). The scope included the population, setting, target audience, intended users and key clinical questions. The key questions provided a framework for the systematic literature review. The scope had a period of open consultation that included direct approach to multiple stakeholder groups (see online supplemental information). The IgAV GDG agreed to present the findings as two guidelines; one for the initial management of IgAV with the intended audience to be general practitioners and general paediatricians (termed guideline 1) and a second guideline for the management of complications associated with IgAV aimed for general paediatricians and paediatric subspecialists (termed guideline 2).

**Table 1:** Topic areas and key questions derived for the systematic review (taken from the UK Kidney Association^99^,^100^)

Topic area	Questions for systematic review
**Initial management (guideline 1**)	
Classification of IgAV	In children and young people with IgAV under the age of 18 years, do classification tools allow professionals to classify IgAV from other clinical diseases to support diagnosis?
Outcome of initial assessment in IgAV	In children and young people with IgAV under the age of 18 years, are there clinical signs and symptoms that indicate urgent complications that require a referral to a specialist?
Nephritis screening in IgAV	In children and young people with IgAV under the age of 18 years, what are the best clinical tests to detect a diagnosis of nephritis?
Treatment of initial presentation of IgAV	In children and young people with IgAV under the age of 18 years, what are the clinical signs and symptoms that indicate the need for therapeutics to manage joint involvement and reduce symptom duration and/or severity?
	In children and young people with IgAV under the age of 18 years, what are the clinical signs and symptoms that indicate the need for therapeutics to manage gastrointestinal involvement and reduce symptom duration and/or severity?
**Management of complications (guideline 2**)	
IgAV associated nephritis	In children and young people under the age of 18 years with IgAV, what are the best investigations to confirm a clinical diagnosis of nephritis?
	In children and young people under the age of 18 years with IgAV, what are the best classification tools to grade a histological diagnosis of IgAV nephritis?
	In children and young people under the age of 18 years with IgAV, what clinical signs, symptoms or investigations define nephritis that requires treatment to reduce the risk of chronic kidney disease?
	In children and young people under the age of 18 years with IgAV, what are acceptable treatments for nephritis to reduce the risk of chronic kidney disease?
IgAV associated gastrointestinal, urological involvement	In children and young people under the age of 18 years with IgAV, what are acceptable treatments for gastrointestinal bleeding that would reduce the duration and/or severity of symptoms?
	In children and young people under the age of 18 years with IgAV, what are acceptable treatments or interventions for the management of intussusception that would reduce the duration and/or severity of symptoms?
	In children and young people under the age of 18 years with IgAV, what clinical signs, symptoms or investigations would support a diagnosis of testicular involvement that requires treatment?
	In children and young people under the age of 18 years with IgAV, what are acceptable treatments or interventions for the management of testicular involvement that would reduce the duration and/or severity of symptoms?
IgAV associated skin involvement	In children and young people under the age of 18 years with IgAV, what clinical signs or symptoms would support for the need to perform a skin biopsy to support a diagnosis?
IgAV persisting, prolonged, recurrent disease	In children and young people under the age of 18 years with IgAV, what clinical signs or symptoms would support a diagnosis of persisting disease?
	In children and young people under the age of 18 years with IgAV, what clinical signs or symptoms would support a diagnosis of recurrent disease?
Long-term follow-up	In children and young people under the age of 18 years with IgAV, what clinical signs or symptoms would indicate the need for follow-up after initial screening to detect long-term complications?

IgAVIgA vasculitis

### Evidence synthesis

A systematic literature review was performed with predefined criteria. Questions about interventions were framed in Patient, Intervention, Comparator, Outcome format. The population were children and young people (aged <18 years) with a confirmed diagnosis of IgAV. The intervention was a classification tool, monitoring or therapeuticmanagement. The comparison was any intervention compared with another. The outcome was related to the diagnosis, complications, duration or severity of symptoms. General concepts were used for the population (“pediatric”, “paediatric”, “child*”, “adolescen*”) and disease (“immunoglobulin A vasculitis”, “IgA vasculitis”, “IgAV”, “Henoch Schonlein purpura”, “HSP”). Concepts were combined using Boolean operators (AND, OR and NOT).^14^ Three bibliographic databases were searched; Medline (https://www.nlm.nih.gov/medline), Embase (https://www.embase.com) and Cochrane Central Register of Controlled Trials (https://www.cochranelibrary.com/central).^15^ The process was performed according to the Cochrane Handbook for Systematic reviews of interventions.^15^ The results were merged, and duplicates were removed. The articles were independently screened and for full-text review according to the eligibility criteria (table 2). Two members synthesised the literature and discussed areas of disagreement. Searching of reference lists was permitted. An evidence table and a narrative summary were produced.

**Table 2:** Eligibility criteria for the literature search

Criteria	Inclusion	Exclusion
**Publication date**	Papers published between 2002 and 2022	Papers published prior to 2002
**Research type**	Primary research	Secondary research
**Study type**	Randomised controlled trials (RCT)If no RCT available to consider;Cohort studiesCase series>5 patientsCase–control studiesMeta-analysisSystematic reviews	Case reportsEditorialsCommentsAnnotationsLettersCommentariesBooks and book chaptersUpdated systematic reviews by same methodology, for example, Cochrane (most recent version will be included)Non-traditional therapies (eg, Chinese medicines) and surgical intervention
**Publication and study status**	PublishedCompleted	UnpublishedOngoing
**Language**	English	Non-English
**Text availability**	Full text available	Full text unavailable

### Generation and grading of recommendations

During the process of achieving group agreement, discussions took into account existing clinical practice and national or international guidelines were evidence was limited or lacking. The modified Grades of Recommendation, Assessment, Development, and Evaluation (GRADE) system was used to evaluate the strength of the recommendation, with grade 1 being a strong recommendation (depicted by ‘we recommend’), and grade 2 being a weaker recommendation (depicted by ‘we suggest’). The quality of evidence was graded A–D with grade A being high-quality evidence, grade B moderate-quality, grade C low-quality evidence from observational studies, or controlled trials with very serious limitations, grade D evidence was generally case studies or expert opinion. Studies were downgraded if there was evidence of bias, indirectness, imprecision or inconsistency of results and the strength of the recommendation could be adjusted taking into context of the balance of benefit or harms to patients. The final draft underwent a period of 4 weeks of open consultation prior to final endorsement.

## Results

### A representative multiprofessional working group

The multiprofessional working group consisted of 28 members including two patient representatives, six general paediatric consultants, one general paediatric trainee, two paediatric surgeons, one paediatric pathologist, one radiologist, three adult nephrologists with expertise in vasculitis, one paediatric gastroenterology trainee, one paediatric emergency consultant, two general practitioners, one medical student, two paediatric rheumatologists, one paediatric gastroenterologist and four paediatric nephrologists. The IgAV GDG met monthly over a 14-month period.

### Generation of the scope document and primary evidence

**Figure 2:**
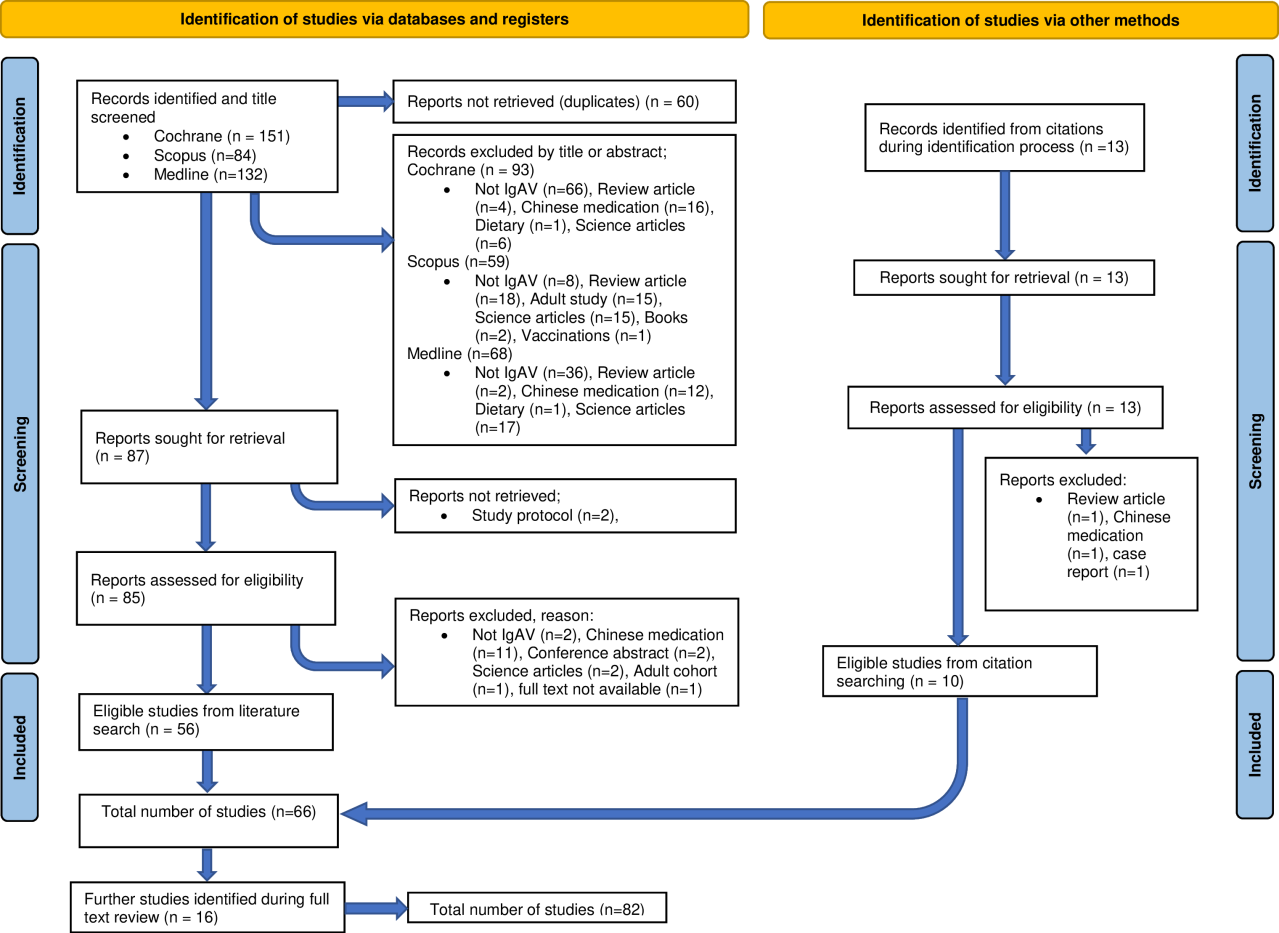
A PRISMA diagram to illustrate the selection of literature for the IgAV recommendations. IgAV, IgA vasculitis; PRISMA, Preferred Reporting Items for Systematic Reviews and Meta-Analyses.

The scope document was distributed to stakeholders and open consultation and feedback was discussed in the monthly working group meeting on 21 January 2022. Following feedback, the GI key questions were united and placed into the complication’s guideline. The systematic literature search was completed on 11 March 2022 and 82 papers were identified. The screening process is illustrated in the Preferred Reporting Items for Systematic Reviews and Meta-Analyses diagram (figure 2) and studies were divided according to topic areas. The initial management guideline had 66 relevant papers of which, 55 related to classification, 16 to specialist referral, 28 to nephritis and 8 to musculoskeletal involvement. The associated complication guideline had 82 relevant papers of which 33 were relevant to established nephritis, 23 to gastrointestinal or urological involvement, 3 to skin, 8 to persisting disease, 34 to recurrent disease and 22 for long-term follow-up. There were no cases of disagreement for inclusion of the papers and 33 papers were helpful for background information however they were ultimately not used to directly inform a recommendation.^16^,^48^

### Recommendations and brief rationale

This article provides a brief summary of the underpinning rationale to support the recommendations (tables3  4). The guidelines are available on the UK Kidney Association website (https://ukkidney.org/renal-association/news/iga-vasculitis-guideline-rcpch-endorsed).

**Table 3:** The recommendations for the initial management of children and young people with IgA vasculitis (IgAV) as taken from The UK Kidney Association^99^

*Number*	Recommendation	*Grade*
***Section 1: Classification of IgAV in clinically suspected disease***		
*1.1*	*We recommend that professionals follow the EULAR/PRINTO/PRES 2010 classification criteria of IgAV.* ^49^	1B
Section 2: Specialist referral in IgAV*		
*2.1*	*We recommend that specialist advice should be sought for children and young people with IgAV and the following clinical presentations (and symptoms or signs):* *Severe gastrointestinal (GI) involvement (definition: unremitting abdominal pain, protein losing enteropathy and/or GI bleeding; sub-specialist: paediatric gastroenterology/paediatric rheumatology*) *Significant nephritis (definition: proteinuria UP: UC ratio of>250 mg/mmol, nephrotic syndrome, nephritic syndrome and/or kidney insufficiency; sub-specialist: paediatric nephrology*)) *Central nervous system (CNS) involvement (definition: cerebral vasculitis presenting as neurological symptoms and/or signs; sub-specialist: paediatric neurology/paediatric rheumatology*) *Pulmonary haemorrhage (definition: pulmonary vasculitis presenting with acute bleeding from the respiratory tract; sub-specialist: paediatric respiratory/paediatric rheumatology*).	1C
*2.2*	*We suggest that specialist advice should be sought for children and young people with IgAV and the following clinical presentations (and symptoms or signs):* *Scrotal and/or testicular involvement (definition: orchiditis; sub-specialist: paediatric surgeon/urology*) *Severe skin manifestations (definition: intense subcutaneous oedema, blistering skin and/or necrotic features; sub-specialist: dermatology/paediatric rheumatology*) *Severe, unremitting, musculoskeletal involvement (definition: arthropathy that requires hospital admission to assist with symptom management and no signs of improvement; sub-specialist: paediatric rheumatology*)	2D
***Section 3: Nephritis screening in IgAV***		
*3.1*	*We recommend that children and young people should have urinalysis testing performed frequently over a period of 6 months to detect a diagnosis of nephritis (for example weekly screening for the first 4–6 weeks then monthly thereafter*).	1B
*3.2*	*We suggest that blood pressure assessment should be performed in children and young people with IgAV at diagnosis and if there is evidence of nephritis*.	2C
***Section 4: Musculoskeletal involvement in IgAV***		
*4.1*	*We recommend appropriate analgesia and rest for musculoskeletal involvement in children and young people with IgAV*.	1C

IgAVIgA vasculitisUP:UCurine protein to urine creatinine ratio

**Table 4:** The recommendations for the management of complications associated with IgA vasculitis (IgAV) in children and young people (taken from The UK Kidney Association^100^)

*Number*	Recommendation	*Grade*
***Section 1: Clinically suspected IgAV nephritis***		
*1.1*	*We recommend that a kidney biopsy is undertaken to confirm a diagnosis of severe nephritis in children and young people with IgAV, where the definition of severe nephritis includes persisting severe proteinuria (UP: UC>250 mg/mmol for up to 4 weeks), persisting moderate proteinuria (UP: UC 100–250 mg/mmol for 3 months*), *AKI stage 1 or greater (at any time), or nephrotic syndrome (at any time; also see Section 2.2*).	1C
***Section 2: Management of histologically proven IgAV nephritis***		
*2.1*	*We recommend that the kidney histology should be classified using the ISKDC classification criteria for children and young people with IgAV*.	1C
*2.2*	*We recommend that a combination of clinical features and the histological ISKDC classification should guide decisions about treatment choices to offer children and young people with IgAV nephritis*.*The following are clinical indications;* *Persisting severe proteinuria (UP: UC>250 mg/mmol for up to 4 weeks*) *Persisting moderate proteinuria (UP: UC 100–250 mg/mmol for up to 3 months*) *AKI stage 1 or greater (serum creatinine>1.5 × previous baseline (if known) or>1.5 × upper limit of normal for age*) *Nephrotic syndrome (clinical signs of oedema, serum albumin<30 g/L, severe proteinuria UP: UC>250 mg/mmol*). *The following are histological indications;* *Class II ISKDC classification with persisting clinical indications* *Class III or above ISKDC classification with clinical indications*	1C
*2.3*	*We suggest that the management of biopsy proven IgAV nephritis should be directed by, or in conjunction with, a paediatric nephrologist*	2B
*2.4*	*We suggest using the following disease-modifying drugs, or a combination of corticosteroids together with a disease-modifying drug, depending on the clinical and histological features in children and young people with biopsy proven IgAV nephritis*.*Corticosteroids;* *Prednisolone 1–2 mg/kg/day (maximum 60 mg/day) for 2–4 weeks then weaned according to clinical response* *In severe nephritis with adverse clinical and histological features (impaired kidney function, nephrotic syndrome, or crescentic features), or where oral absorption is potentially compromised, intravenous methylprednisolone 10–30 mg/kg (maximum of 1 g/day) once daily for three consecutive days may be used followed by the use of oral prednisolone*. *Disease-modifying drug (listed in alphabetical order);* *Azathioprine* *Calcineurin inhibitors (ciclosporin or tacrolimus*) *Cyclophosphamide* *Mycophenolate mofetil*. *Rapidly progressive glomerulonephritis is managed more aggressively with preference for intravenous treatment*.	2B
*2.5*	*We suggest that in cases of IgAV nephritis with persisting proteinuria (UP: UC>100 mg/mmol for 3 months or UP: UC>50 mg/mmol for 6 months) the use of an angiotensin converting enzyme inhibitor (ACEi) or an angiotensin receptor blocker (ARB) should be considered as adjunctive or monotherapy under the guidance of a nephrologist, even if they haven’t met the threshold for performing a kidney biopsy (section 1.1*).	2C
***Section 3: Management of acute GI bleeding***		
*3.1*	*We suggest that corticosteroids (prednisolone 1–2 mg/kg/day for 1–2 weeks) are considered for children and young people with IgAV within 3 days of onset of severe abdominal pain (defined as pain requiring hospital admission) or acute GI bleeding after appropriate clinical review and the exclusion of other causes including intussusception*.	2B
***Section 4: Management of suspected or proven intussusception***		
*4.1*	*We recommend that specialist surgical and(/or)radiological advice is sought for children and young people with IgAV and abdominal symptoms suggestive of intussusception*.	1C
***Section 5: Management of suspected or proven testicular involvement***		
*5.1*	*We suggest that testicular involvement (orchiditis) should be considered in boys with IgAV who develop painful scrotal oedema that is associated with palpable purpuric lesions*.	2D
*5.2*	*We suggest that treatment with corticosteroids (prednisolone 1–2 mg/kg/day for 1–2 weeks) should be considered in boys with IgAV who develop orchiditis after appropriate specialist advice such as a surgical opinion has been sought*.	2D
***Section 6: Management of cases with atypical features***		
*6.1*	*We suggest that a skin biopsy is undertaken in children and young people with IgAV who have an atypical purpuric/petechial rash or to exclude alternative diagnoses*.	2B
*6.2*	*We suggest that when a skin biopsy is performed the histological analysis should specifically include evaluation of IgA deposition using immunofluorescence (fresh specimen) or immunohistochemistry (fixed tissue*).	2C
***Section 7: Definition of persisting or recurrent disease***		
*7.1*	*We suggest that children and young people with IgAV and a typical purpuric/petechial rash persisting for more than 1 month should be defined as having persisting disease*.	2B
*7.2*	*We suggest that children and young people with IgAV who present with a reappearance of the typical purpuric/petechial rash after a symptom-free period of greater than 1 month should be defined as having recurrent disease*.	2B
***Section 8: Long term follow up in IgAV***		
*8.1*	*We recommend that children and young people with IgAV should have follow up whilst there is evidence of nephritis and for at least 3 years if they have experienced biopsy proven nephritis*.	1C

AKIacute kidney injuryISKDCInternational Study of Kidney Diseases in ChildrenUP:UCurine protein to urine creatinine ratio

### Initial management

#### Disease classification

European Alliance of Associations for Rheumatology (EULAR)/Paediatric Rheumatology INternational Trials Organisation (PRINTO)/Paediatric Rheumatology European Society (PRES) Classification criteria were agreed by the IgAV GDG to classify IgAV. This was based on a study published by Ozen *et al*^49^ involving 860 children, demonstrating excellent sensitivity and specificity when used to distinguish it from other forms of vasculitis. It formed a strong recommendation and aligned with the SHARE consortium.^13^

#### Early specialist referral

No studies were identified that adequately addressed indications for specialist referral. Therefore, studies related to the need for hospital admission and/or treatment were used to generate discussion and create two recommendations. Severe organ involvement was felt to be so significant that if missed, there may be patient harm and assigned as a strong recommendation.

#### Nephritis screening

There were no studies directly comparing different clinical tests for the detection of nephritis. A total of 28 studies reported the use of clinical tests, including urinalysis (20/28 studies; 71%), a combination of urinalysis and other tests (7 studies; 25%) or renal function alone (1 study; 4%). In view of the lack of evidence, the group concluded that urinalysis was the minimal acceptable standard for nephritis screening incorporating existing clinical practice.

#### Treatment of musculoskeletal involvement

There were no specific studies found that robustly evaluated treatments for joint involvement. Five reports included analgesia, symptomatic support, hydration and bed rest. Prednisolone, given at a high dose over 4 weeks, was evaluated in a randomised controlled trial of 171 children where it reduced pain score and duration of pain, although the duration was not statistically significant.^50^ Due to the lack of long-term musculoskeletal morbidity related to this condition, the IgAV GDG agreed that the limited evidence did not justify the use of high-dose corticosteroids due to the side effect profile.

### Management of complications

#### IgAV nephritis

The clinical indications for conducting a kidney biopsy were adapted from the SHARE initiative as this was felt to be the strongest evidence available. The ISKDC classification criteria were used in 21 studies. Koskela *et al* specifically compared the ISKDC classification criteria with the modified semiquantitative score suggesting superiority in predicting renal outcomes.^51^ The IgAV GDG were aware that studies in this area were ongoing and agreed that the ISKDC criteria were the most widely accepted in the literature and clinical practice at the time of deriving the recommendations. This is likely to be a priority area for the future update. To determine treatment initiation, the histological classification grade alone was used by most studies (9/13; 69%). No studies made direct comparisons. A further two studies (2/13; 15%) used a combination of histological features and clinical features,^52^ which aligned with the European SHARE initiative recommendation.^13^ A combination of clinical and histological features was agreed to be most reflective of clinical practice.

Aligned with previous Cochrane reviews, no studies provided conclusive evidence of a preferred treatment option for biopsy-proven nephritis. Treatments alluding to benefit included corticosteroids,^1113 17 52^,^70^ cyclophosphamide,^11 13 52 53 55 56 59 60 62 65 68 70 71^ azathioprine,^11 13 53 55 56 64 68 70^ ciclosporin A,^11 52 58 63 72^ mycophenolate mofetil,^11 13 52 70^ tacrolimus,^52 67^ intravenous immunoglobulin,^54 69^ urokinase,^60^,^62^ triptolide,^66^ low molecular-weight heparin,^73^ plasma exchange,^62 74^ warfarin,^61 62^ dipyridamole.^61 62 65^ The quality of studies was weak with heterogeneity and a short list of potential agents was developed.

The use of renin-angiotensin aldosterone system inhibition (RAASi) was reviewed in six low-quality studies.^1113 53 55^,^57^ As RAASi is standard of care for children with proteinuria, this was a specific recommendation.

### Gastrointestinal involvement

For severe GI involvement, 16 studies reported positive efficacy from corticosteroids.^1339 50 59 75^,^86^ This included one placebo controlled randomised trial (n=171 participants) demonstrating a statistically significant reduction in the severity of abdominal pain within 2 weeks from diagnosis compared with placebo.^50^ A systematic review by Weiss *et al* found a statistically significant benefit from abdominal pain within 24 hours following the use of corticosteroids.^87^ Weiss *et al* and Zhao *et al* demonstrated a decreased risk of intussusception, abdominal surgery, endoscopy and imaging if corticosteroids were received within 72 hours.^88 89^ Due to short-term morbidity, the IgAV GDG concluded that on balance, the evidence was sufficient to consider the use of a short course of corticosteroids for severe abdominal pain and/or GI bleeding, once intussusception had been excluded. Specific investigations for GI bleeding were beyond the scope.

### Intussusception management

There were 11 studies regarding intussusception management (10/23; 43%).^5976 78 79 84 87^,^91^ Weiss *et al*^88^ described the most common abdominal surgery, which was intra-abdominal small bowel manipulation.^87 88^ No studies compared interventions and the quality of the evidence was weak. Due to the high risk of complications, the IgAV GDG agreed on a strong recommendation to obtain specialist advice.

### Testicular involvement

Reports on testicular involvement were limited with orchiditis, defined as painful, acute scrotal oedema associated with palpable purpuric lesions.^79^ Five small studies described treatment,^13 75 79 81 85^ including the effective use of corticosteroids (a total of 15 patients in three studies).^79 81 85^ The literature highlighted that surgical exploration may be required to exclude differential diagnoses.^75^ The group agreed that a short course of corticosteroids could be considered for this complication and this aligned with the European SHARE initiative.^13^

### Atypical disease course

Three studies (3/83; 3.6%) included performing a skin biopsy in atypical disease and it forms part of the classification criteria, therefore it was recommended.

### Persisting or recurrent disease

Eight studies (8/82; 9.8%) included a definition for persisting disease and agreed on skin lesions for >1 month and 34 provided a definition for recurrent IgAV with the majority (n=19/34; 56%) using rash reappearance after a 1-month symptom-free period.

### Long-term follow-up

There were 22 studies (22/82; 27%) with indications for long-term follow-up,^1719 39 52 55 56 61 63 66 68 70 71 75 82 84^,^86 92^ of which 17 related to nephritis.^1952 55 56 61 63 66 68 70 71 75 84^,^86 93 94 96^ Based on low-quality evidence, the IgAV GDG made a strong recommendation to follow-up patients who had experienced biopsy-proven nephritis due to the risk of CKD.

## Discussion

IgA vasculitis is a small vessel vasculitis characterised by IgA1-dominant immune deposits. Due to the recognised risk of kidney failure that is consistently reported at 1%–2% in children, and far greater at 2.9%–23.9% in the adult population, there is a major unmet need for evidence generation for this condition.^6 97^ The aim of this project was to use a best available evidence, group agreement, based approach to develop national recommendations for the initial management of IgAV and its associated complications in children and young people. Using accredited methodology and an inclusive, multiprofessional, representative working group, evidence was generated based on key topics. Recommendations for both the initial management, aimed at primary and secondary care colleagues, and the management of complications, aimed at secondary and tertiary care colleagues, for IgAV in children and young people have been established. These guidelines have been endorsed nationally and are available online.

The derived recommendations were developed from primary evidence, incorporating pooled expertise where evidence was lacking, and largely aligned with existing international consensus management such as that published by the SHARE initiative.^13^ Similarities to this previous work included the accepted classification criteria, screening for nephritis and broadly the management of gastrointestinal and musculoskeletal symptoms. Topic areas that required added detail or were new included when to consider a specialist referral, kidney biopsy indications and nephritis management, the use of RAASi and the definitions of recurrent or persisting disease.

Guidelines form an important component to guide equity of treatment for rare diseases across regions and facilitate standardisation as a pathway for clinical trials. While suitable targets for this disease may not yet be apparent as the underlying pathophysiology remains incompletely understood, the reported mechanisms leading to IgAV nephritis are strikingly similar to those described in IgA nephropathy with galactose-deficient IgA1 acting as a key antibody for immune complex formation, there therefore may be repurposing opportunities.^98^ These first national guidelines also include parameters for audit and highlight research priorities, including defining the rates of hypertension; the indications, timing and duration of corticosteroids for GI involvement; suitable GI outcome measures and GI biomarkers; management of persisting and/or recurrent disease; and predictors and treatments for biopsy-proven nephritis. This study does have limitations, which includes the significant lack of high-quality evidence to generate strong recommendations together with areas of actively evolving research that may be subjected to change in the near future, for example, the optimal method to score the kidney histology, and finally the national based approach when this disease has unmet need for children worldwide. However, this manuscript demonstrates a successful method to unite clinical approaches despite these recognised limitations in a disease with a severe paucity of evidence. Future updates will continue to incorporate emerging evidence and reflect changes in clinical management.

## Conclusion

IgAV is a rare condition that predominates in children where there is huge unmet need in evidence-based management. These guidelines provide an important foundation to standardise management nationally, which should reduce variability in clinical care ensuring equity of access for all children. Indirectly it is also anticipated that they will be a catalyst to enhance the generation of evidence.

## supplementary material

10.1136/archdischild-2024-327364 online supplemental file 1

## Data Availability

No data are available.
