# Supplementary material for: National recommendations for the management of children and young people with IgA vasculitis: a best available evidence, group agreement-based approach
Source: Arch Dis Child. 2024 Oct 8;110(1):e327364. doi: 10.1136/archdischild-2024-327364 (PMC11671997; doi:10.1136/archdischild-2024-327364)
Supplement: online supplemental file 1 [file archdischild-110-1-s001.pdf]

Supplementary information

1. A list of stakeholder groups that were directly given the opportunity to feedback on the scope document

**Stakeholder**
British society of Paediatric gastroenterology, hepatology and nutrition (BSPGHAN)
British Society of Rheumatology (BSR)
Association of Paediatric Emergency Medicine APEM
Royal College of Emergency Medicine RCEM
Paediatric Emergency Medicine research collaborative for the UK and Ireland (PERUKI)
British association of paediatric surgery BAPS
Royal College of General Practitioners (RCGP)
British Society of Paediatric radiology BSPR
Royal College of Pathologists
British association of general paediatrics
General and adolescent research collaboration UK and Ireland (GAPR-UKI)
British Society of Paediatric Dermatology (BSPD)
Kidney care UK
Vasculitis UK
Versus arthritis UK
Barbara Ansell Network (BANNAR)
